# IgA pemphigus as an immune checkpoint inhibitor-associated skin manifestation

**DOI:** 10.1016/j.jdcr.2024.02.025

**Published:** 2024-03-08

**Authors:** Tristan V.M. Bruijn, Anne Geraedts, Carmen A. Vlahu, Lies.H. Jaspars, Yannick S. Elshot

**Affiliations:** a Department of Dermatology, The Netherlands Cancer Institute - Antoni van Leeuwenhoek, Amsterdam, Netherlands; b Department of Dermatology, Amsterdam UMC, University of Amsterdam, Amsterdam, Netherlands; c Department of Pathology, The Netherlands Cancer Institute - Antoni van Leeuwenhoek, Amsterdam, Netherlands; d Department of Pathology, Amsterdam UMC, University of Amsterdam, Amsterdam, Netherlands

**Keywords:** anti-PD-1, drug eruption, IgA, immune checkpoint inhibitor, immune-related adverse event, pembrolizumab, pemphigus

## Introduction

The introduction of antiprogrammed cell death protein 1 monoclonal antibodies into clinical practice has dramatically transformed the prognosis of patients with metastatic melanoma and several other advanced-stage malignancies.^1^ Despite significant clinical benefits, side effects from this activated immunological antitumor response are expected with a unique spectrum of immune-related adverse events (irAEs). The skin is the earliest and most prevalent affected site, as cutaneous irAEs have been reported in more than one-third of treated patients. The most common clinical presentations include pruritus, maculopapular, and lichen planus-like eruptions.^2^ Although less common, immunobullous manifestations such as bullous pemphigoid and severe blistering eruptions are increasingly reported.^3^, ^4^, ^5^ Herein, we report a case of a patient who developed IgA pemphigus after initiating treatment with pembrolizumab.

## Case report

An 81-year-old woman was referred to the outpatient dermatology clinic with a painful pustular rash on the scalp and axilla. She had been previously treated for metastatic stage IV nonsmall cell lung carcinoma with pembrolizumab and pemetrexed for over 2 years resulting in stable remission without any cutaneous adverse events. After 6 months of treatment interruption, pembrolizumab monotherapy was reintroduced owing to progressive disease. Within 2 weeks, she developed painful and pruritic pustules that merged into crusted plaques on the scalp and a few painful pustules in the armpits. No other drugs had recently been initiated, and the concurrent medication consisted of omeprazole. The patient’s medical history included chronic obstructive pulmonary disease and stage IA (AJCC 8) breast carcinoma, for which she underwent wide local excision without adjuvant therapy. There was no history of dermatological disease. Following a negative skin swab result, minocycline treatment was initiated.

**Fig 1:**
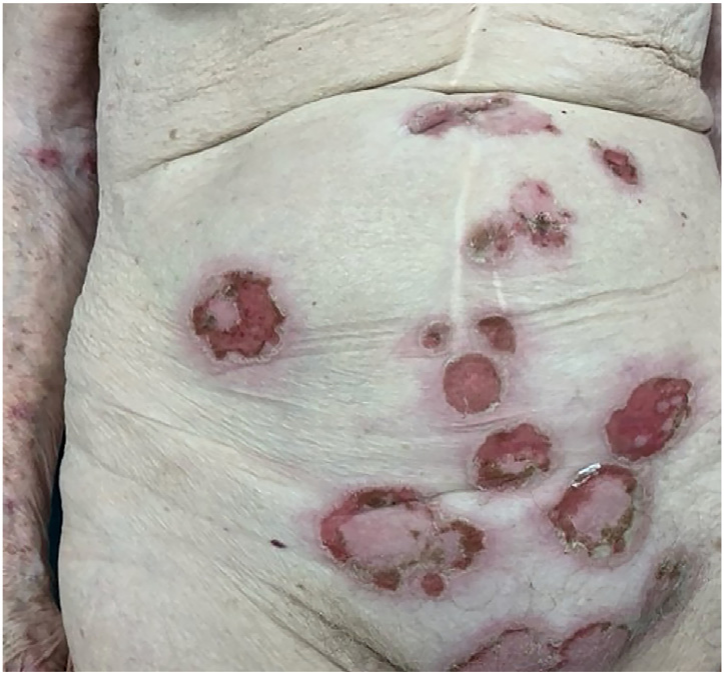
Clinical pictures of the trunk and extremities showing flaccid vesicles and pustules arranged in erythematous annular plaques with crusted erosions.

**Fig 2:**
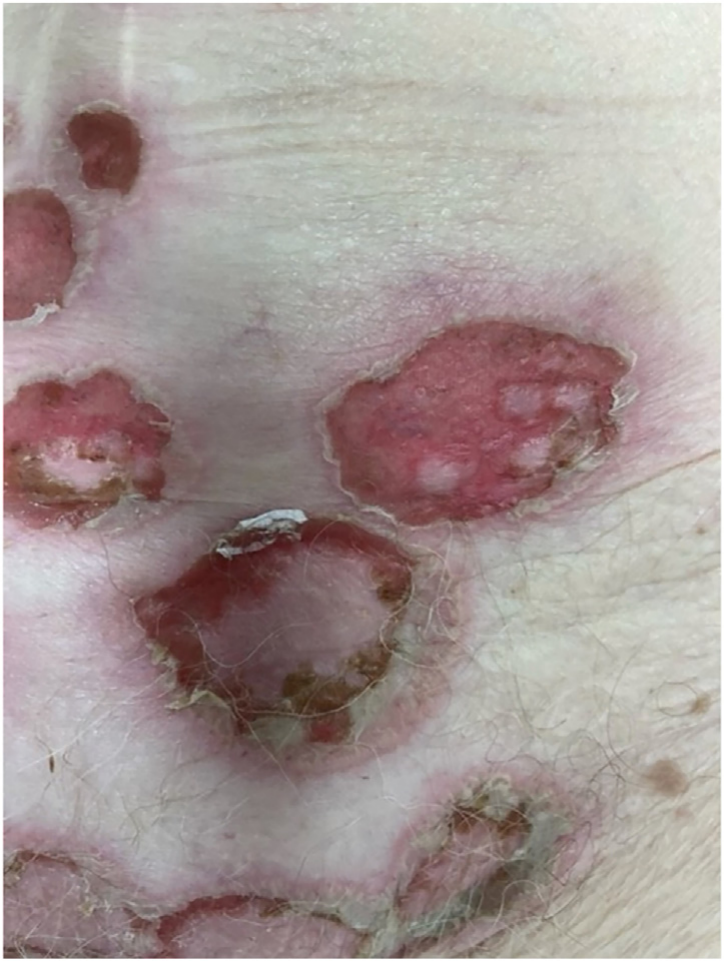
Detailed picture of erythematous annular plaques with crusted erosions.

**Fig 3:**
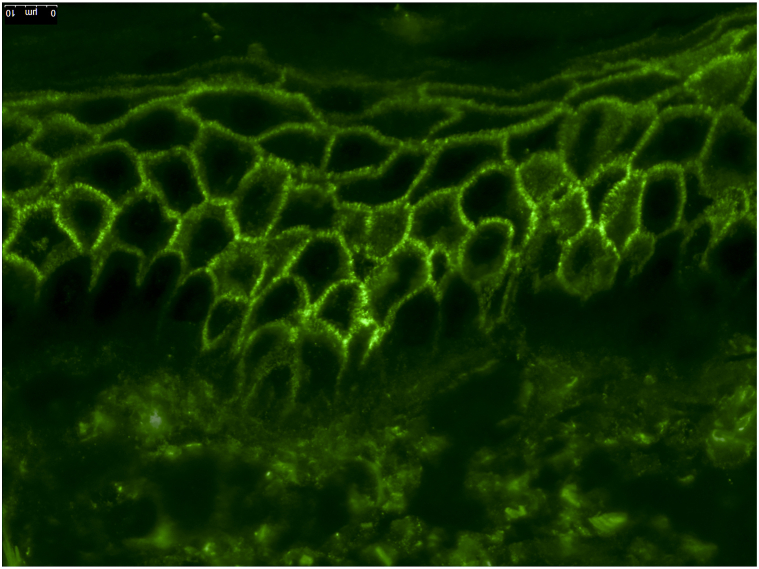
Direct immunofluorescence revealed IgA deposits in the intercellular space of the epidermis (“ics pattern”) of the perilesional skin (fluorescein isothiocyanate, ×400).

Two weeks later, she presented with fever, rapid progression of the skin eruptions, and severe pruritus. Physical examination revealed several flaccid vesicles and pustules arranged in erythematous annular plaques with crusted erosions mainly located on the trunk and extremities without mucosal involvement (Figs 1 and 2). Owing to the severity of her symptoms (grade 3, CTCAE Version 5.0), pembrolizumab was interrupted, and the patient was started on oral prednisolone 40 mg daily, which halted the progression of the blisters. A lesional punch biopsy was performed, revealing a mostly reepithelialized intraepidermal pustule with various secondary changes. In the corner of the pustule, focal acantholytic suprabasal splitting was noted, but the overall picture lacked diagnostic clarity. Nevertheless, direct immunofluorescence of the perilesional skin exhibited granular depositions of IgA in an intercellular space staining—or “chicken wire” pattern (Fig 3), indicative for the diagnosis of IgA pemphigus. Indirect immunofluorescence results were negative. However, this procedure was performed after the initiation of immunosuppressive treatment. Dapsone 100 mg daily was introduced with planned prednisolone tapering over 3 months. Complete resolution of cutaneous symptoms was achieved after 4 weeks. During this period, a partial response of the metastatic nonsmall cell lung carcinoma was observed, prompting the reinitiation of pembrolizumab following prednisolone tapering. However, before pembrolizumab could be reintroduced, the patient experienced disease progression and ultimately died following best supportive care.

## Discussion

Here, we report a rare case of IgA pemphigus following pembrolizumab treatment. While most cutaneous irAEs associated with antiprogrammed cell death protein 1 checkpoint inhibitors are classified as low-grade, bullous eruptions tend to lead to a higher patient symptom burden and possible discontinuation of therapy. While bullous pemphigoid is the most common type of immunobullous dermatosis, other types such as lichen planus pemphigoid, mucous membrane pemphigoid, and linear IgA bullous dermatosis are scarcely reported.^3^, ^4^, ^5^

IgA pemphigus is a rare autoimmune bullous disorder characterized by painful and pruritic pustular or vesiculobullous skin eruptions that result from epidermal IgA deposition. Circulating IgA antibodies attack (non)desmosomal keratinocyte cell surface components responsible for cell-to-cell adherence and induce an inflammatory reaction, resulting in neutrophilic infiltration and accumulation in the epidermis with subsequent blistering and pustule formation.^6^ IgA pemphigus is usually divided into 2 distinct subtypes: subcorneal pustular dermatosis type with IgA limited to the upper dermis (targeting desmosomal protein desmocollin-1) and intraepidermal neutrophilic dermatosis type with widespread distribution of IgA throughout the epidermis (usually targeting desmoglein-1 or desmoglein-3). The clinical presentation may overlap between the 2 subtypes, generally showing flaccid vesicles and pustules that rupture to form annular or circinate confluent erythematous plaques with crusts and are predominantly distributed on the trunk, extremities, and intertriginous areas. Mucosal or palmoplantar involvement is rarely observed. Generalized pruritus is reported in most cases (>60%), while systemic symptoms, such as fever, vomiting, or malaise, are typically absent.^6^

Direct immunofluorescence and indirect immunofluorescence are currently the gold standard for diagnosing IgA pemphigus. Histological features include subcorneal or intraepidermal pustules with variable degrees of acantholysis and usually a mixed inflammatory cell infiltrate. Unlike other bullous dermatoses, systemic corticosteroids alone do not often sufficiently control IgA pemphigus.^6^ Dapsone, originally an antibiotic used to treat leprosy. It is often the drug of choice for primary treatment because it has dual antimicrobial and neutrophilic antiinflammatory effects. In dermatology, these properties are helpful in treating various dermatologic diseases, including chronic inflammatory diseases, autoimmune bullous eruptions, and eosinophil- and neutrophil-mediated disorders.^7^ As in our case, successful treatment of IgA pemphigus typically results in the complete resolution of skin lesions without residual scarring.

IgA pemphigus is associated with lymphoproliferative and autoimmune disorders. However, drug-induced IgA pemphigus is rare, with only anecdotal case reports linking this uncommon disease to thiol drugs or adalimumab.^8^^,^^9^ In the oncologic setting, a case of imatinib-induced subcorneal pustular dermatosis was published by Sinha et al.^10^

In conclusion, we present a case of IgA pemphigus with probable causal association with pembrolizumab. Causality assessment was carried out using Naranjo Adverse Drug Reaction Probability scale, resulting in a score of 5, indicating a probable correlation. While late onset (>1 year) immune-related cutaneous adverse events have been described,^2^^,^^3^ an alternative explanation could be a (antiprogrammed cell death protein 1) luxated paraneoplastic syndrome, as indicated by the subsequent disease progression. However, to our knowledge, no paraneoplastic cases in solid tumors have been reported. As immune checkpoint inhibitors are increasingly used, oncology healthcare providers should be familiar with the range of bullous irAEs to identify and treat them appropriately without discontinuation of immune checkpoint inhibitor treatment.

## Conflicts of interest

None disclosed.

## References

[1] Alsaab H.O., Sau S., Alzhrani R. (2017). PD-1 and PD-L1 checkpoint signaling inhibition for cancer immunotherapy: mechanism, combinations, and clinical outcome. Front Pharmacol.

[2] L’Orphelin J.M., Cassecuel J., Kandolf L. (2023). European Association of Dermato-Oncology. Cutaneous manifestations induced by check point inhibitors in 120 melanoma patients - The European MelSkinTox study. J Eur Acad Dermatol Venereol.

[3] Kawsar A., Edwards C., Patel P. (2022). Checkpoint inhibitor-associated bullous cutaneous immune-related adverse events: a multicentre observational study. Br J Dermatol.

[4] Siegel J., Totonchy M., Damsky W. (2018). Bullous disorders associated with anti-PD-1 and anti-PD-L1 therapy: a retrospective SKIN TOXICITIES OF ICIs | 85 analysis evaluating the clinical and histopathologic features, frequency, and impact on cancer therapy. J Am Acad Dermatol.

[5] Ingen-Housz-Oro S., Milpied B., Badrignans M. (2022). Severe blistering eruptions induced by immune checkpoint inhibitors: a multicentre international study of 32 cases. Melanoma Res.

[6] Kridin K., Patel P.M., Jones V.A., Cordova A., Amber K.T. (2020). IgA pemphigus: a systematic review. J Am Acad Dermatol.

[7] Ghaoui N., Hanna E., Abbas O., Kibbi A.G., Kurban M. (2020). Update on the use of dapsone in dermatology. Int J Dermatol.

[8] Kishimoto K., Iwatsuki K., Akiba H., Motoki Y., Kaneko F. (2001). Subcorneal pustular dermatosis- type IgA pemphigus induced by thiol drugs. Eur J Dermatol.

[9] Saunder M.B., Glassman S.J. (2013). Palmoplantar subcorneal pustular dermatosis following adalimumab therapy for rheumatoid arthritis. Int J Dermatol.

[10] Sood A., Sinha P., Raman D.K., Sinha A. (2018). Imatinib-induced IgA pemphigus: subcorneal pustular dermatosis type. Indian Dermatol Online J.

